# FASN Protein Overexpression Indicates Poor Biochemical Recurrence-Free Survival in Prostate Cancer

**DOI:** 10.1155/2020/3904947

**Published:** 2020-06-18

**Authors:** Zhi Cao, Yalong Xu, Fei Guo, Xi Chen, Jin Ji, Huan Xu, Jingyi He, Yongwei Yu, Yinghao Sun, Xin Lu, Fubo Wang

**Affiliations:** ^1^Department of Urology, Changhai Hospital, Navy Medical University, Shanghai, China; ^2^Department of Pathology, Changhai Hospital, Navy Medical University, Shanghai, China

## Abstract

**Backgrounds:**

Fatty acid synthase (FASN) has been regarded as a prognostic marker in prostate cancer (PCa). In this study, we evaluated FASN expression at both mRNA and protein levels and assessed the association between FASN expression and prognosis in male Han Chinese with PCa treated with radical prostatectomy (RP).

**Methods:**

Expression profile and prognostic value of FASN were analyzed in tissue microarray (TMA) and data retrieved from databases including TCGA public database, GEO database, and our sequencing data with whole clinicopathological characteristics.

**Results:**

FASN expression was associated with clinical parameters and biochemical recurrence of prostate cancer. The relative expression of FASN mRNA was higher in the tumor tissue in all public databases and our sequencing data (*p* < 0.001). A similar result was seen in tissue microarray (TMA) (*p* < 0.001). Analysis of our sequencing data indicated that FASN's relative expression was associated with tumor stage (*p* = 0.048), and FASN expression was positively associated with the Gleason score (*p* = 0.004) and seminal vesicle invasion (*p* = 0.011) in TMA. We found that high FASN expression was an independent predictor of shorter BCR-free survival with univariate and multivariate survival analysis (*p* < 0.05), rendering FASN an optimal prognostic biomarker in male Han Chinese with prostate cancer.

**Conclusions:**

Our study demonstrated that FASN was overexpressed at mRNA and protein levels in PCa. We found that patients with high FASN expression had a shorter BCR-free survival, showing its value as a prognostic biomarker in male Han Chinese with PCa.

## 1. Introduction

Prostate cancer (PCa) ranks 2nd in male tumor morbidity and 5th in mortality worldwide [1]. According to the latest report, the annual growth rate of PCa morbidity and mortality in China is as high as 7.2% and 5.5%, respectively, making it the fastest-growing tumor in China [2]. Early PCa was confined to the capsule; radical prostatectomy (RP) or radiotherapy is often recommended [3]. However, although most patients respond to the treatments initially, a large portion of them will progress to recurrence and/or metastasis. The percentage of PCa patients who undergo radical prostatectomy experiencing biochemical recurrence (BCR) is approximately 25% [4, 5]. Therefore, timely and accurate estimation of BCR risk for patients with poor prognosis, especially for those who need adjuvant treatment, is of utmost importance to improve outcomes. The present clinical prognostic parameters, such as prostate-specific antigen (PSA), Gleason score (GS), and clinical or pathological tumor stage, with limitations in the differentiation of the biological heterogeneity of tumors, are unable to accurately estimate the risk of aggressive prostatic tumors. Therefore, the identification of a novel sensitive and specific biomarker to monitor the prognosis of PCa is urgently needed.

As we know, a significant hallmark of PCa is abnormal lipid metabolism, as first observed by Medes and colleagues in 1953 [6]; actually, a lipogenic phenotype is a distinctive feature of PCa cells. Fatty acids (FAs) are the essential constituents for energy metabolism, which is consistent with exogenous FAs (from diet) and endogenous FAs (synthesized *in vivo*). Adequate energy to support uncensored growth and proliferation of cancer cells relies on the utilization of FAs by their *β*-oxidation to generate ATP, while exogenous FA uptake from the diet alone cannot meet the energy demands. Accordingly, FAs generated through de novo FA synthesis play a pivotal role in cancer tissue with a 270 kDa key enzyme named Fatty acid synthase (FASN), which is involved in the process of transformation of acetyl-CoA and malonyl-CoA to FAs that are minimally expressed in many normal tissues except liver and adipose tissue [7], as well as tumor tissue. In fact, FASN is often overexpressed in both the early (prostatic intraepithelial neoplasia) and late (metastasis) stages of PCa, suggesting that it is involved in the process of the initial phases of prostate tumorigenesis, maintenance, and biological aggressiveness [8–10]. It is considered that FASN is a *bona fide* oncogene based on its high expression in prostate cancer and its effect in protecting cancer cells from apoptosis [11]. Several studies have shown that the inhibition of FASN expression induces apoptosis in multiple types of tumors, including PCa. De Schrijver et al. reported that silencing FASN with siRNA significantly inhibited LNCaP cell growth and ultimately led to apoptosis [12]. Kridel et al. also demonstrated that the novel FASN inhibitor Orlistat significantly inhibited proliferation, migration, and invasion of PC-3 tumor cells and induced cell apoptosis in mouse xenograft models [13], which has also been demonstrated both *in vivo* and *in vitro* lately by Migita et al. [14]. Moreover, increased expression of FASN is significantly related to poor prognosis, which means it may be used as a prognostic biomarker for PCa [15, 16]. It has also been reported that the expression of FASN can predict the Gleason score and pathological stage [16, 17]. However, all these studies were mainly done in the western population. No literature has reported its role in male Han Chinese. Thus, in the present study, we explored the expression profile of FASN and its prognostic value by database retrieval and immunohistochemistry in male Han Chinese.

## 2. Method and Materials

### 2.1. Gene Expression Analysis

We obtained TCGA prostate cancer gene expression data from the University of California Santa Cruz (UCSC) Cancer Genomics Browser, which contained 495 prostate cancer cases and 52 paracancerous controls with whole clinicopathological characteristics. The sequencing data of our research center involved 65 prostate cancer cases with their cancerous and matched paracancerous normal tissue. In addition, an independent GEO database (GSE46602, GSE6752) was also downloaded to extract information on the mRNA expression of FASN for further analysis.

### 2.2. Patients Cohort for the Construction of the Tissue Microarray (TMA)

A total of 188 patients who had undergone radical prostatectomy at the Department of Urology, Changhai Hospital, from October 2002 to December 2008 were eligible for this study. Their archived tissues from the Department of Pathology were used to construct TMA. None of these patients were treated preoperatively with hormonal or radiation therapy. Patients' medical records were accessible for the clinical and pathological information. Two pathologists observed the prostatectomy specimens and evaluated them according to the College of American Pathologists [16] without knowing patients' clinical outcomes or follow-up data. All 188 cases' pathological stages were classified according to the 2002 staging criteria of the American Joint Committee on Cancer (AJCC). This protocol was approved by our institutional medical ethics review committee.

### 2.3. Follow-Up

All patients' serum PSA levels were evaluated every 3 months in the first year, every 6 months from the second year to the fifth year, and annually thereafter. Their follow-up data were accessible by consulting the hospital medical records and calling to patients or their family members. We defined BCR as the sustained elevation of the serum total PSA level above 0.2 ng/ml for at least twice. Overall biochemical recurrence-free survival was defined as the time from the date of surgery to the date of BCR (first detection of PSA level above 0.2 ng/ml).

### 2.4. TMA Construction

As we described previously, TMA was constructed with formalin-fixed paraffin-embedded tissue blocks from prostate cancer patients who underwent radical prostatectomy [18]. The slides were selected by two pathologists originally with cancerous tissue and matched paracancerous normal tissue without inflammatory zones that colored with different dyes. Then, the TMA was cut into 3 mm sections and stained with hematoxylin–eosin to ensure that the cores adequately represented the diagnostic areas.

### 2.5. Immunohistochemistry and Evaluation of Immunostaining

The expression of FASN was detected by immunohistochemistry (IHC) using a commercial FASN antibody (dilution 1 : 200; Abcam, Cambridge, UK). The IHC assay was conducted according to the manufacturer's instructions with all the staining reagents. The evaluation of IHC staining was performed by two independent pathologists without knowing the patients' clinical information using a modified histological score method based on both the percentage of positively stained cells and the intensity of staining. Under 40× visual field magnification, the intensity score (I) of staining was classified into four grades (ranks 0 to 3), while the extent score (E) of stained cells was classified into five grades (ranks 0 to 4). The IE score was constructed as follows: intensity score (I) × extent score (E), with a maximum score of 12. If the interobserver variability exceeded 15%, the sample sides would be rescored to reach a consensus for each patient's slide. We arbitrarily defined IE score (0-5) as low expression and IE score (6-12) as high expression.

### 2.6. Statistical Analysis

SPSS software version 21.0 (IBM Corp., Armonk, NY, USA) was utilized for the statistical analysis. FASN expression (high expression vs. low expression) between different PSA levels, Gleason score, and pathological grading was evaluated by the Kruskal–Wallis *H*-test. Associations between FASN expression and histological subtypes and clinical variables (surgical margin, seminal vesicle invasion, and the pathological lymph node category) were analyzed using the Mann–Whitney *U*-test for categorical variables. The association between FASN expression levels and risk of BCR following RP was analyzed by the Kaplan-Meier estimator and log-rank tests. Moreover, all the independent factors (age, PSA level, Gleason score, TNM categories, and FASN expression level) were further tested using Cox's proportional hazard regression for multivariate comparison as the outcome. Differences were considered statistically significant with *p* < 0.05.

## 3. Results

### 3.1. FASN mRNA Expression in the Database

**Figure 1:**
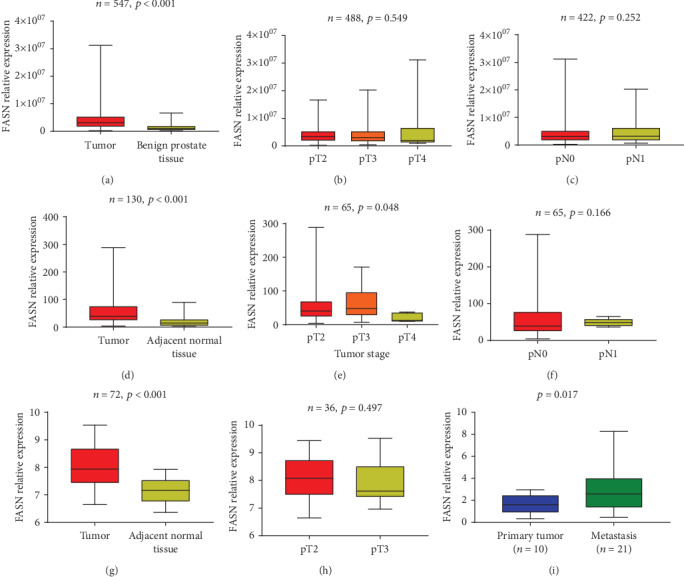
mRNA expression of FASN in TCGA public database (a–c), our sequencing data (d–f), GSE46602 (g, h), and GSE6752 (i). FASN expressions comparison between tumor and benign prostate tissue (a, d, g). FASN relative expression pattern among lymph node categories (c, f) and tumor stages (b, e, h). Comparison of FASN expression in primary prostate tumor tissue and metastasis (i) (note: T2: tumor stage 2; T3: tumor stage 3; T4: tumor stage 4; pN0: pathology lymph nodes 0; pN1: pathology lymph nodes 1).

The heatmaps of differential genes including FASN in different databases could be seen in Supplementary Figure 1, as well as LogFC forms of the differential genes (Supplementary Table 1, Supplementary Table 2, and Supplementary Table 3). In the TCGA database, we found that the relative expression of FASN mRNA was significantly higher in tumor tissue compared to benign tissue (*p* < 0.001, Figure 1(a)). However, FASN relative expression was not associated with tumor stage (*p* = 0.549, Figure 1(b)) or pathological lymph node status (*p* = 0.252, Figure 1(c)). A similar result was observed in our sequencing data and GSE46602: FASN mRNA expression in tumor tissue was relatively high (*p* < 0.001, Figure 1(d); *p* < 0.001Figure 1(g)), but FASN relative expression was not associated with tumor stage in GSE46602 (*p* = 0.497, Figure 1(h)), while in our sequencing data PCa in pT3 showed higher expression of FASN (*p* = 0.048, Figure 1(e)). Furthermore, our results indicated that PCa with metastasis expressed higher FASN (*p* < 0.017, Figure 1(i)).

### 3.2. Patients Description in TMA

The clinical and pathological characteristics, which enrolled 188 patients with a mean age of 61.03 ± 6.84 years in the study, are summarized in Table 1.

### 3.3. FASN Expression in TMA

**Figure 2:**
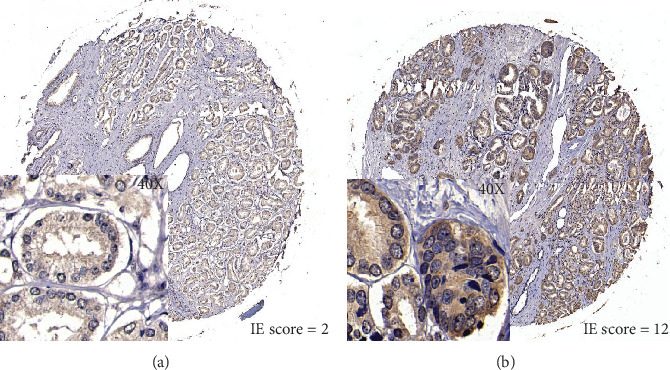
FASN expression pattern was analyzed by the IE score in prostate cancer tissues (40 times zoom in square).

**Figure 3:**
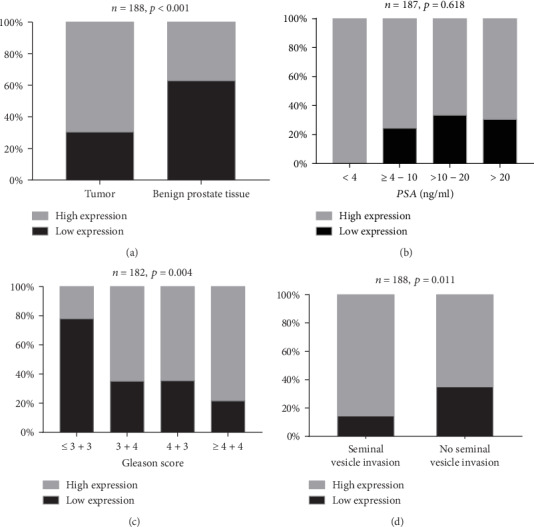
FASN expression in TMA by IHC. Comparison between tumor tissue and benign tissue (a). The relationship between FASN expression and preoperation PSA (b), Gleason score (c), and seminal vesicle invasion (d).

In our study, FASN was expressed wildly in both the prostatic epithelial cells and paracancerous normal tissue. We used the IE score to measure the expression of FASN protein. Representative FASN IE score patterns are shown in Figure 2. For further analysis, we defined a cut-off value of IE score of 5 after calculating the average IE score, of which ≥6 was regarded as high expression. The relationship of FASN expression and clinicopathological parameters was calculated in Table 2. The staining of FASN in tumor tissue was significantly stronger than in paracancerous normal tissue (*p* < 0.001, Figure 3(a)). There was no difference in FASN expression among different preoperative PSA levels (*p* = 0.618, Figure 3(b)), pathological tumor stages (*p* = 0.569), pathological lymph node categories (*p* = 0.294), or surgical margin status (*p* = 0.187). FASN expression was positively associated with Gleason score (*p* = 0.004, Figure 3(c)) and seminal vesicle invasion (*p* = 0.011, Figure 3(d)).

### 3.4. Association between FASN Expression and BCR

**Figure 4:**
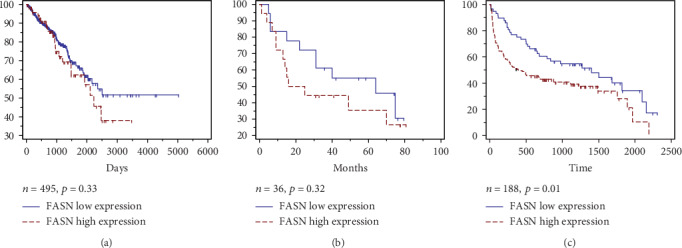
The relationship between FASN expression and patient prognosis. (a) Kaplan–Meier analysis of BCR-free survival in patients' data retrieved from TCGA (*n* = 495, *p* = 0.33), (b) Correlation between FASN expression and BCR-free survival in GEO dataset (*n* = 36, *p* = 0.32). (c) Disease-free survival in TMA patients (*n* = 188, *p* = 0.01). The log-rank test shows that PCa patients with high FASN expression have shorter disease-free survival time.

The follow-up data were extracted from the GEO database (GSE46602), in which 22 of 36 patients experienced BCR after an average postoperative follow-up duration of 23.9 ± 22.2 months. It seems that low expression of FASN had an association with longer BCR-free survival, while the log-rank test indicated that there were no significant differences (*p* = 0.33, Figure 4(a)). The same result was found in the TCGA database, with a nonsignificant association between FASN expression and BCR (*p* = 0.32, Figure 4(a)). On the other hand, 188 patients' follow-up information was available in TMA, which included 114 patients who experienced BCR with a postoperative follow-up duration of 13.7 ± 18.1 months. In these patients, the log-rank test indicated that patients with higher FASN expression had shorter BCR-free survival (*p* = 0.01, Figure 4(c)). In the univariate Cox proportional hazards regression analyses, FASN expression was associated with BCR after radical prostatectomy (*p* = 0.011). After adjusting for some clinicopathological parameters, multivariate Cox analysis revealed that FASN expression (*p* = 0.004), preoperative PSA level (*p* = 0.002), Gleason score (*p* < 0.001), and pathological lymph node category (*p* < 0.001) were all associated with recurrence (Table 3).

## 4. Discussion

Cancer cell metabolism is quite distinctive in that DNA and protein synthesis are increased due to overconsumption of energy [19, 20]. Another hallmark is increased de novo FA synthesis related to the glycolytic pathway, which promotes cell growth, survival, and drug resistance. Moreover, continuous synthesis of de novo FAs is required to provide lipids as the raw material for membrane production and energy metabolism in highly proliferating cancer cells [21]. Many malignant carcinomas, including PCa, behave in the same lipid metabolism patterns involving the pivotal enzyme FASN. A more important fact is that further study demonstrated that accumulated cholesteryl ester is associated with prostate cancer aggressiveness [22]. Pelton et al. also reported that the evaluated level of circulating cholesterol was positively linked to the development of prostate cancer [23]. Actually, an analysis included 22 separate studies from the Oncomine public database revealed that FASN mRNA expression, as well as other lipid metabolic enzyme genes, is increased in PCa [24], which is consistent with the results of our study. Of these, both databases and immunohistochemistry results showed high FASN mRNA expression in prostate cancerous tissue compared to paracancerous normal tissue.

It has been demonstrated that FASN acts as an oncogene involved in the intrinsic pathway of apoptosis. Migita et al. reported an inverse relationship between FASN expression and the apoptotic rate in human PCa samples and transgenic mice expressing FASN showed an increased rate of proliferation and decreased rate of castration-induced apoptosis in the prostate [14]. De Schrijver et al. showed that attenuated cancer cell growth and induced apoptosis were positively associated with the downregulation of FASN expression mediated by siRNA [12]. Ample evidence has revealed that FASN may act as a biomarker in promoting tumor progression, including cell proliferation, cell adhesion, migration, and invasion, as well as pseudopodia formation, which plays a critical role in PCa proliferation and metastasis [25, 26]. Moreover, androgen, which has been proven to be related to PCa progression, regulates FASN expression and its activity in human prostate cancer cell lines by activating the expression of ubiquitin-specific protease-2a (USP2a), which stabilizes FASN expression to prolong PCa cell survival [27]. In contrast, increased FASN expression promoted PCa cells to resist apoptosis as the tumor progressed to androgen independence, which rendered the gene a metabolic oncogene [28, 29]. However, the heterogeneity of FASN expression among individuals has been observed in many studies. Genomic amplification of FASN has been reported in approximately one-fourth of patients with PCa [9]. Rossi et al. evaluated FASN expression levels in 64 patients with primary PCa by immunohistochemistry and showed that FASN expression is weakly in more than half of patients, while strongly in 8% and moderately in 30% [10]. Similarly, Migita et al. reported that gene expression varied among individuals [14]. Because the overexpression of FASN in PCa is significantly heterogeneous, it is supposed that FASN is a prognostic biomarker [15].

It has been reported that FASN upregulation participated in the progression of PCa, which is the initial event in PCa development and increased substantially from prostatic intraepithelial neoplasia to low grade, to high grade, and to androgen-independent bone metastases [10]. Our TMA data also indicated that FASN expression was positively related to the Gleason score (*p* = 0.004), which is consistent with a previous study [30]. The mechanism was proposed as the result of activation and nuclear localization of Akt and cytoplasmic stabilization of *β*-catenin, subsequently affecting membranal function and antiapoptotic proteins [14, 31].

In addition, we analyzed the prognostic value of FASN in predicting BCR in patients who undergo RP using public database and immunohistochemistry. The results of BCR-free survival time of the TCGA and GEO databases indicated a correlation between high FASN expression and shorter disease-free survival, albeit statistically nonsignificant. In contrast, the analyses of our TMA data subsequently illustrated that low expression of FASN was associated with longer BCR-free survival, as reported by Shurbaji et al. [15] in 1996, which demonstrated FASN's potential prognostic role in PCa progression. We proposed that there may be no ethical difference in FASN expression in PCa, as our data indicated a similar pattern in Han Chinese patients as the foreign population (TCGA and GEO databases). Thus, we believe that FASN may act as a prognostic biomarker. In the univariate logistic analysis, significant effects of the pT category to predict BCR were observed, while not observed in the multivariate analysis, which could be explained as the difference contributing to the included factors. In addition, FASN expression was found significantly both in univariate analysis and multivariate analysis, high FASN expression was identified as a significant factor to predict poor BCR and PCa progression. In spite of the differences in samples and ethnicity of the three datasets, we could still draw a conclusion of the oncogenic effect of FASN in PCa.

Our study still has several limitations. First, it was limited in sample size as a single-center study. Second, our study focused only on RNA and protein levels, more studies are needed to explore its potential mechanisms. Furthermore, our follow-up time was relatively short compared with other cohorts.

## 5. Conclusion

Our study demonstrated that FASN was overexpressed at mRNA and protein levels in PCa. We found that patients with high FASN expression had a shorter BCR-free survival, showing its value as a prognostic biomarker in male Han Chinese with PCa.

## Figures and Tables

**Table 1:** The clinical and pathological characteristics of patients.

Variable	*n*	Percentage (%)
Age (year)		
<50	4	2.1
50-60	23	12.2
60-70	82	43.6
≥70	76	40.4
NA	3	1.6
Preoperative PSA level (ng/ml)		
<4	2	1.1
4-10	37	19.7
10-20	69	36.7
>20	79	42.0
NA	1	0.5
Gleason score		
≤3 + 3	9	4.8
3 + 4	60	31.9
4 + 3	34	18.7
≥4 + 4	79	43.4
NA	6	3.2
pT category		
pT2a–2b	34	18.1
pT2c	79	42.0
pT3a-b	71	37.8
pT4	4	2.1
pN category		
pN0	121	64.4
pN+	32	17
NA	35	18.6
Surgical margin		
Negative	106	56.4
Positive	82	43.6
Capsular invasion		
Yes	68	36.2
No	120	63.8
Seminal vesicle invasion		
Yes	42	22.3
No	146	77.7
Nerve invasion		
Yes	80	42.6
No	108	57.4

**Table 2:** FASN expression status in TMA.

Variable	No. of successfully analysed samples	FASN immunohistochemistry result		
		Low	High	*p*
Tissue type	188			<0.001
Prostate cancer	188	57	131	
Paracancerous tissue	188	118	70	
Preoperative PSA level (ng/ml)	187			0.618
<4	2	0	2	
4-10	37	9	28	
10-20	69	23	46	
≥20	79	24	55	
Gleason score	182			0.004
≤3 + 3	9	7	2	
3 + 4	60	21	39	
4 + 3	34	12	22	
≥4 + 4	79	17	62	
pT category	188			0.569
pT2a–2b	34	12	22	
pT2c	79	25	54	
pT3a-b	71	18	53	
pT4	4	2	2	
pN category	153			0.294
pN0	121	38	83	
pN+	32	7	25	
Seminal vesicle invasion	188			0.011
Yes	42	6	36	
No	146	51	95	
Surgical margin	188			0.187
Negative	106	28	78	
Positive	82	29	53	

**Table 3:** Univariate and multivariate analyses of survival of FASN expression.

	Univariate analysis		Multivariate analysis	
	HR (95% CI)	*p* value	HR (95% CI)	*p* value
FASN (high/low)	1.717 (1.132-2.604)	0.011	2.005 (1.248-3.221)	0.004^∗^
PSA level (>10/≤10)	3.060 (1.675-5.589)	<0.001	2.903 (1.486-5.669)	0.002^∗^
Gleason score (≥8/<8)	3.122 (2.125-4.587)	<0.001	2.375 (1.493-3.778)	<0.001^∗^
pT category (T3+T4/T2)	2.951 (2.023-4.305)	<0.001	1.503 (0.912-2.477)	0.110
pN category (pN1/pN0)	4.083 (2.573-6.477)	<0.001	3.024 (1.846-4.953)	<0.001^∗^
Age (>70/≤70)	0.792 (0.531-1.181)	0.253		

## Data Availability

Answer: No. Comment: The data used to support the findings of this study are available from the corresponding author upon request.

## Supplementary Materials

Supplementary Materials Supplementary Figure 1: the heatmap of the differential genes in TCGA (A), GSE46602 (B), and GSE6752 (C). Supplementary Table 1: the LogFC form of the differential genes in TCGA. Supplementary Table 2: the LogFC form of the differential genes in GSE46602. Supplementary Table 3: the LogFC form of the differential genes in GSE6752.
