# Supplementary figures and images for: FASN Protein Overexpression Indicates Poor Biochemical Recurrence-Free Survival in Prostate Cancer
Source: Dis Markers. 2020 Jun 18;2020:3904947. doi: 10.1155/2020/3904947 (PMC7321525; doi:10.1155/2020/3904947)

## Slide 1
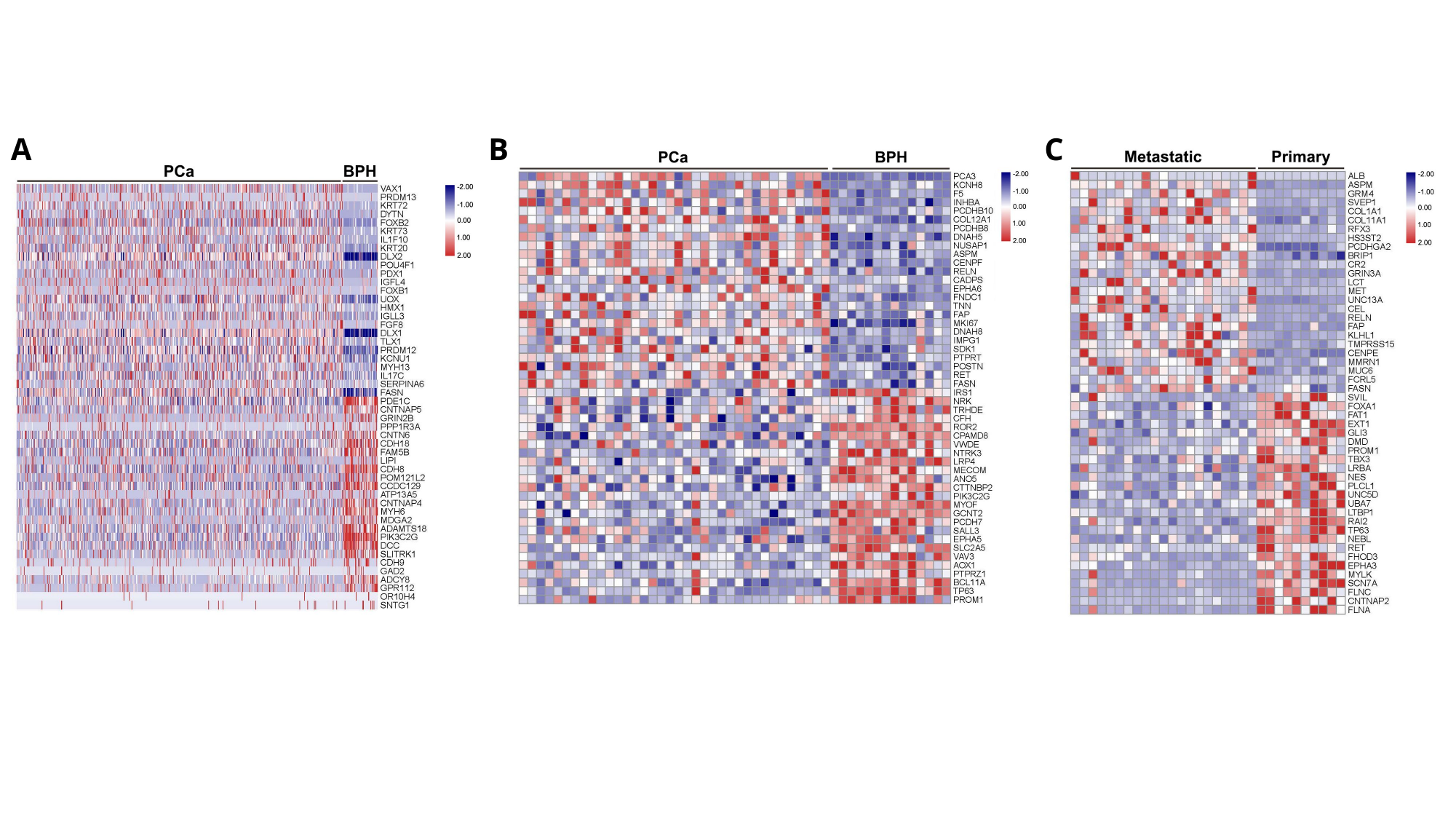

A
B
C

Supplement: Supplementary Materials — Supplementary Figure 1: the heatmap of the differential genes in TCGA (A), GSE46602 (B), and GSE6752 (C). Supplementary Table 1: the LogFC form of the differential genes in TCGA. Supplementary Table 2: the LogFC form of the differential genes in GSE46602. Supplementary Table 3: the LogFC form of the differential genes in GSE6752. [file 3904947.f1.zip › 3904947.f1/Supplemental Figure-1.pptx]
